# Preparation and Properties of Highly Elastic, Lightweight, and Thermally Insulating SiO_2_ Fibrous Porous Materials

**DOI:** 10.3390/ma15093069

**Published:** 2022-04-23

**Authors:** Yitian Li, Anran Guo, Xiaojing Xu, Yunjia Xue, Liwen Yan, Feng Hou, Jiachen Liu

**Affiliations:** 1 Key Lab of Advanced Ceramics and Machining Technology of Ministry of Education, School of Materials Science and Engineering, Tianjin University, Tianjin 300072, China; liyt@tju.edu.cn (Y.L.); xueyj@tju.edu.cn (Y.X.); lwyan@tju.edu.cn (L.Y.); fhou@tju.edu.cn (F.H.); 2 Aerospace Business Department, China Academy of Launch Vehicle Technology, Beijing 100076, China; xjxu@tju.edu.cn

**Keywords:** quartz fibers, sodium hexametaphosphate dispersant, porous materials, thermal insulation, compression resilience

## Abstract

Fibrous porous materials are one of the most commonly used high-temperature insulation materials because of their high porosity and low thermal conductivity. Due to their wide applications in the aerospace and energy industries, the investigation of high-elastic thermally insulating porous materials has attracted increasing attention. In order to improve the elasticity of fibrous porous materials, quartz fibers with high aspect ratio were used as matrix, sodium hexametaphosphate (SHMP) was selected as dispersant. We innovatively reported that a unique three-dimensional skeleton structure was constructed by adjusting the dispersion of fibers in the slurry, and the lightweight, thermal insulating and elastic SiO_2_ fibrous porous material was then prepared by the compression molding method. The characterization results of zeta potential and absorbance showed that the addition of SHMP was an effective method to enhance the dispersibility of quartz fibers in the slurry. SiO_2_ fibrous porous materials with 0.4 wt% SHMP content exhibited an ideal three-dimensional skeleton structure, which endowed the porous material with high porosity (89.39%), low density (0.04751 g/cm^3^), and low thermal conductivity (0.0356 W·m^−1^·K^−1^). The three-dimensional skeleton structure formed by overlapping fibers with high aspect ratios endowed the porous material with excellent elasticity. SiO_2_ fibrous porous materials with 0.4 wt% SHMP content could undergo large strains of 30% and achieved a resilience ratio of 81.69% under the 30th compression cycle. Moreover, after heat treatment at 800 °C, SiO_2_ fibrous porous materials also maintained good elasticity with a resilience ratio of more than 80%.

## 1. Introduction

Fibrous porous materials are mainly composed of oxide fibers, which overlap with each other, forming a stable three-dimensional skeleton structure [1,2,3]. This three-dimensional skeleton structure provides this material with high porosity, low density, and low thermal conductivity [4,5]. Therefore, fibrous porous materials are regarded as promising high-temperature insulating materials in the flue gas filtration, aerospace, and energy industries [6,7]. Due to the expansion of applications, the preparation and properties of elastic fibrous porous materials have attracted extensive attention [8,9].

Hou et al. [10] successfully prepared porous materials with low thermal conductivity (0.19–0.22 W·m^−1^·K^−1^) and high compressive strength (3–13 MPa) by a TBA-based gel casting process using mullite fibers with an aspect ratio of 40–75 as the matrix. However, the resilience ratio of the material was only 66–70% at an applied strain of 2%. To further improve the elasticity of fibrous porous materials, researchers have proposed using flexible fibers with a high aspect ratio as a matrix to construct a three-dimensional skeleton structure with a higher resilience ratio [11,12,13]. Wang et al. [14] prepared TiO_2_ nanofiber material with a density of 0.4 mg/cm^3^ by a blow-spinning process. The materials exhibited excellent elasticity with a resilience ratio of 95% at 400 °C after 10 cyclic compression tests at an applied strain of 23%. However, the above-mentioned methods (TBA-based gel casting process and blow-spinning process) are not applicable to mass production in the industrial field due to the complex processes and high cost. The thermal insulation and elastic properties of the materials prepared by the above method also need to be improved.

Compression molding is an ideal process to fabricate porous fibrous materials with a three-dimensional skeleton structure, because it can realize large-scale molding and mass production by low cost and simple equipment [15,16,17]. The main process consists of the following two parts: dispersing fibers in an aqueous solution to prepare fiber slurries and pouring the slurry into a mold to fabricate a three-dimensional skeleton structure by applying pressure. In this process, the dispersibility of fiber is one of the key factors affecting the uniformity and deformability of the three-dimensional skeleton structure, which further affects the elasticity of fibrous porous materials. However, due to the hydrophobicity, fibers with high aspect ratios are more prone to entanglement and agglomeration in aqueous solutions, which will result in a decrease in the dispersibility of fibers [18,19]. At present, the addition of dispersants is considered to be an effective method to modify the dispersibility of fibers in slurries [20]. Akbar et al. [21] reported that the addition of carboxymethylcellulose nano (CMC) could lead to the uniform dispersion of carbon fibers in the slurry and found that an increase in the CMC solution pH value enhanced the dispersibility of carbon fiber. However, after reaching the critical concentration of 0.8%, a further increase in CMC content led to the formation of large carbon fiber agglomerates. Chen et al. [22] compared the effects of hydroxyethyl cellulose (HEC), hydroxypropyl methyl cellulose (HPMC) and sodium hexametaphosphate (SHMP) on the dispersibility of glass fibers. The results showed that the glass fibers exhibited the greatest dispersibility in the slurry with the addition of 0.012% HPMC. Lü et al. [23] prepared carbon fiber-reinforced shells using hydroxypropyl methylcellulose (HPMC) as the dispersant. The results suggested that the addition of HPMC could improve the dispersibility of carbon fibers, enhancing the strength of the fiber-reinforced shell. However, excess HPMC increased the viscosity of the solvent, affecting the liquidity of the slurry and reducing the strength of the shell. Therefore, selecting an appropriate dispersant is an effective method to improve the dispersibility of fibers in the slurry. Based on the properties of quartz fibers and the operability of the compression molding, we selected an anionic dispersant, sodium hexaphosphate, to investigate its influence on the dispersibility of fiber slurry.

In this work, quartz fibers with high aspect ratio were used as a matrix, sodium hexametaphosphate (SHMP) was selected as dispersant. We innovatively proposed to construct unique three-dimensional skeleton structure by adjusting the dispersion of fiber slurries, and to prepare highly elastic, lightweight and heat-insulating SiO_2_ fibrous porous materials by using compression molding method. Fiber slurries were prepared using sodium hexametaphosphate (SHMP) as the dispersant, and the effect of SHMP content on the dispersibility of quartz fibers in slurry was investigated. The influences of the dispersibility of fibers on the three-dimensional skeleton structure, physical properties, and elasticity of SiO_2_ fibrous porous materials were further studied. Moreover, the thermal insulation performance of porous materials at different heat treatment temperatures was analyzed.

## 2. Materials and Methods

### 2.1. Fabrication Process

**Figure 1:**
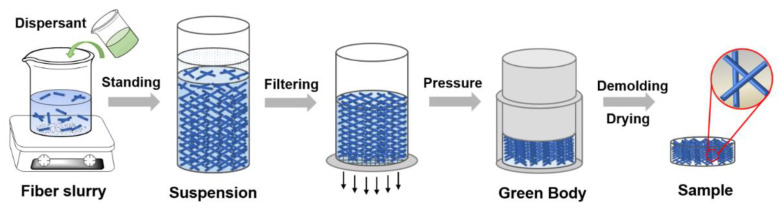
Fabrication process of SiO_2_ fibrous porous materials.

Figure 1 shows the fabrication process of SiO_2_ fibrous porous materials. In this study, quartz fiber (3–5 μm in diameter, purity 99.95%, Hubei Feilihua Quartz Glass Co., Ltd., Jingzhou, China) was used as the matrix, and sodium hexametaphosphate (SHMP, Aladdin Industrial Co., Ltd., Shanghai, China) was used as the dispersant to prepare SiO_2_ fibrous porous materials. The fibers were pretreated using a high-speed pulper (2890 r/min, 20 s) to obtain quartz fibers with an aspect ratio of 500–800. Then, distilled water, quartz fibers and SHMP were mixed to prepare fiber slurries with a fiber content of 0.2 wt%, and the pH of the slurry was adjusted to 3.0–3.5 using H_2_SO_4_ and NaOH pure reagent. The well-stirred fiber slurry was poured into a special mold of Φ100 × 50 mm size and left to stand for 1 h to obtain a suspension slurry. Then, after the fiber block was formed by filtering the suspension slurry, a pressure of 0.1 MPa was applied to the fiber block to prepare a green body. After demolding, the green body was dried in an oven at 65 °C for 12 h to obtain a porous material with a size of φ100 × 20 mm. To investigate the effect of dispersion on various properties of porous materials, fiber slurries with different SHMP contents (0.1–0.7 wt%) and the corresponding SiO_2_ fibrous porous materials were prepared in this experiment.

### 2.2. Characterization

**Figure 2:**
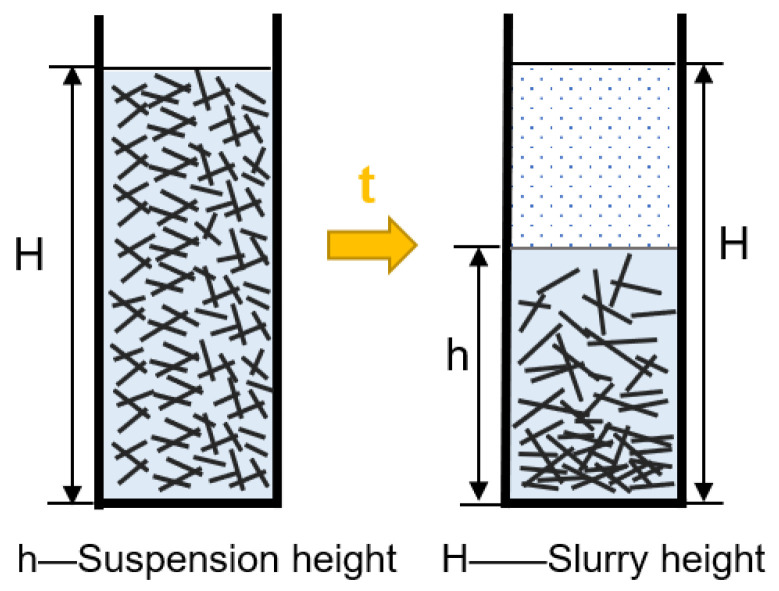
Schematic diagram of the suspension ratio for slurry.

The zeta potential of the quartz fiber slurry was tested by a zeta potential analyzer (Malvern Zetasizer Nano ZS, Malvern, UK). The suspension ratio was calculated by measuring the ratio of suspension height h to slurry height H, as shown in Figure 2. The absorbance of the quartz fiber slurry was tested by a UV spectrophotometer (UV1802G, Tianjin Guanze Co., Ltd., Tianjin, China). The wavelength of maximum absorbance of quartz fiber slurry (λ_Max_) is 968 nm. According to the Lambert–Beer law [24], the absorbance of the slurry can be expressed as:

(1) $$A=\mathrm{lg}\left(\frac{I}{I_{0}}\right)=-\mathrm{lg}T$$

where *A* is the absorbance, *T* is the transmittance, *I*_0_ is the incident light intensity, and *I* is the transmitted light intensity. When light shines into the quartz fiber slurry, the fibers absorb and reflect the incident light, resulting in the transmitted light intensity *I* being much lower than the incident light intensity *I*_0_. Well-dispersed quartz fibers do not undergo significant entanglement and agglomeration in the slurry, which benefits light transmission. Therefore, slurries with good fiber dispersibility exhibit higher absorbance in the test results.

The micromorphology of SiO_2_ fibrous porous materials was observed using a Zeiss hot-field scanning electron microscope (SEM, Sigma 300, Oberkochen, Germany). The phase transition of the samples was investigated by X-ray diffraction (XRD, Rigaku D/Max2500, Tokyo, Japan). The sample bulk density was calculated based on the geometric dimension and the mass of the porous materials. The sample true density was measured with a Micromeritics AccuPyc 1330 gravimeter. The sample porosity was calculated by:

(2) $$P=\left(1-\frac{\rho }{\rho_{0}}\right)\times 100\%$$

where *P* is the porosity of the sample, *ρ* is the sample bulk density, and *ρ*_0_ is the true density of the sample. The thermal conductivity of the porous materials was investigated by a thermal constant analyzer (Hot disk TPS 2500S, Uppsala, Sweden). The compressive properties of the samples were tested by an electronic universal testing machine (CMT4303, Meister Industrial Systems, Shenzhen, China) with a loading speed of 0.5 mm/min. The backside temperatures of the samples were detected by a heating furnace (KSL1700X, Hefei Kejing Co., Ltd., Hefei, China) to analyze thermal insulation performance.

## 3. Results

### 3.1. Dispersibility of Quartz Fibers

**Figure 3:**
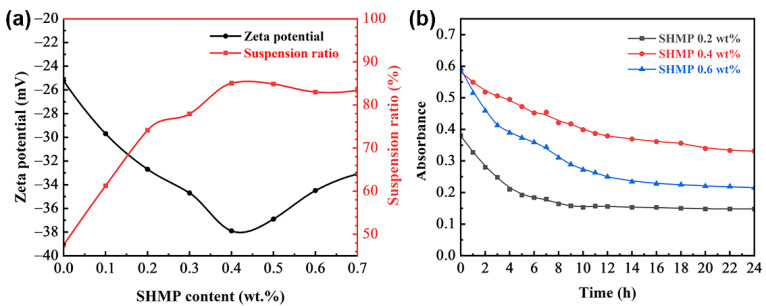
(**a**) Zeta potential and suspension ratio of quartz fiber slurries with different SHMP contents. (**b**) Absorbance of quartz fiber slurries with different SHMP contents at different standing times.

**Figure 4:**
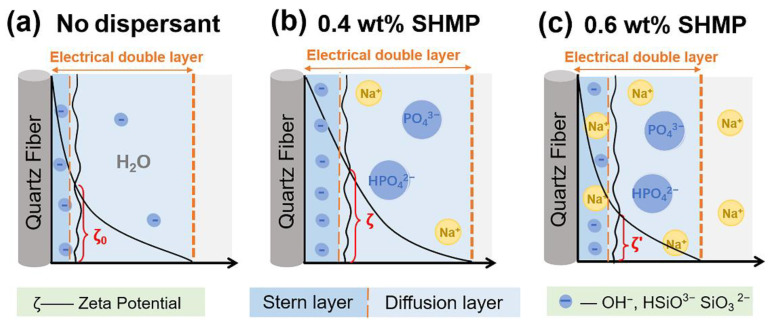
Schematic mechanism of the zeta potential of fiber slurries with different SHMP contents. (**a**) 0, (**b**) 0.4 wt% and (**c**) 0.6 wt%.

The addition of inorganic salt dispersants to the fiber slurry can change the charge distribution on the fiber surface, resulting in a structural change in the corresponding electrical double layer, which can be reflected by the change in zeta potential [25]. Figure 3a shows the effect of SHMP content on the zeta potential of the fiber slurry. The zeta potential is mainly influenced by the charge of the Stern layer in the electrical double layer [26]. As shown in Figure 4a, the aqueous fiber slurry produced large amounts of OH^−^, HSiO^3−^ and SiO_3_^2−^ ions by hydrolysis and ionization. These anions were closely adsorbed in the Stern layer on the surface of quartz fibers. Therefore, as shown in Figure 3a, the zeta potential (ζ_0_) of the fiber slurry without dispersant addition was negative (−25.23 mV). After the addition of dispersant, SHMP was hydrolyzed, resulting in the formation of a large amount of PO_4_^3−^ and HPO_4_^2−^ anions. This increase in negative charge concentration would lead to the adsorption of more anions by the Stern layer, which resulted in the expansion of the electrical double layer between adjacent fibers [27]. Thus, the absolute value of the zeta potential of the slurry increased from 25.23 to 29.7 mV as the SHMP content increased from 0 to 0.1 wt%. When the SHMP content was 0.4 wt%, the absolute value of the zeta potential reached a maximum of 37.9 mV. However, as the SHMP content continued to increase, as shown in Figure 4c, the amount of Na^+^ ions (counter ions) increased, and the counter ions in the diffusion layer diffused into the Stern layer under electrostatic repulsion. This phenomenon led to the compression of the electrical double layer, resulting in a decrease in the absolute value of the zeta potential (ζ’) [28,29]. Thus, when the SHMP content increased from 0.4 to 0.7 wt%, the absolute value of the zeta potential of the fiber slurry decreased from 37.9 to 33.1 mV.

The suspension ratio refers to the ratio of the height of the slurry after standing for a period of time to the initial slurry height [30]. It is often used to evaluate the dispersion of solid particles in the slurry. Figure 3a shows the suspension ratios of the quartz fiber slurries with different SHMP contents after standing for 24 h. When the SHMP content increased from 0 to 0.4 wt%, the suspension ratio increased from 48% to 85%. This is mainly because the addition of SHMP increased the zeta potential of the slurry, thus improving the dispersibility of the quartz fibers. However, when the SHMP content increased from 0.4 to 0.7 wt%, there was no significant change in the suspension ratio. The zeta potential analysis showed that the dispersion of the fiber decreased with increasing SHMP content in this content range. Therefore, the absorbance of the fiber slurry was further used in this experiment to evaluate the dispersibility of fibers. Figure 3b shows the absorbance of the fiber slurries with SHMP contents of 0.2 wt%, 0.4 wt% and 0.6 wt% at different standing times. For the same standing time, the absorbance of the slurry with 0.4 wt% SHMP content were consistently greater than the absorbance of the other two slurries, indicating that the fibers were more uniformly dispersed in the slurry with 0.4 wt% SHMP content. The results of this analysis coincided with those of the zeta potential analysis in Figure 3a. In summary, the dispersibility of fibers in the slurry increased and then slightly decreased with an increase in SHMP content, and the slurry with 0.4 wt% SHMP content exhibited superior dispersibility of quartz fiber.

### 3.2. Characterization of SiO_2_ Fibrous Porous Materials

**Figure 5:**
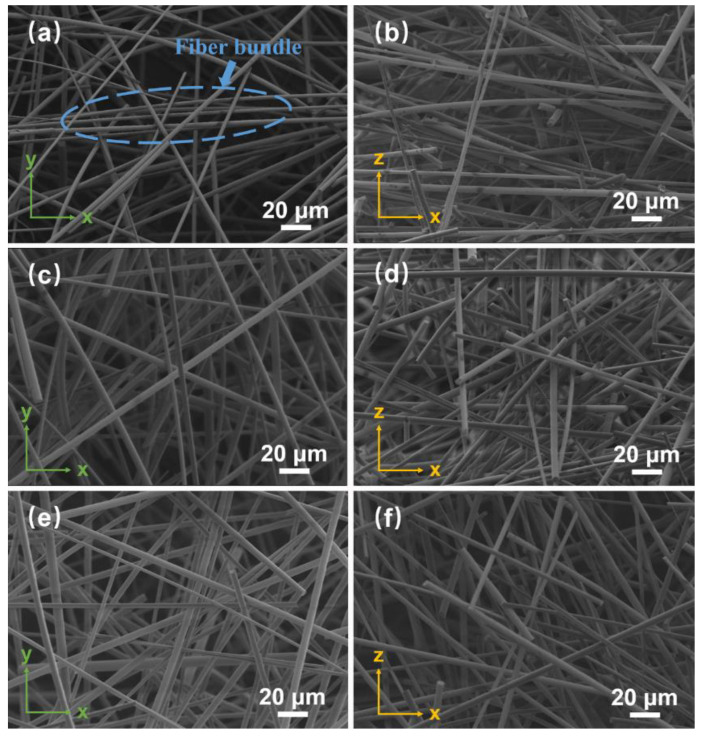
SEM images of SiO_2_ fibrous porous materials with different SHMP contents. (**a**,**b**) 0.2 wt%, (**c**,**d**) 0.4 wt% and (**e**,**f**) 0.6 wt%.

During the compression molding process, the quartz fibers in the suspension slurry are closely lapped by the applied pressure, thus forming a three-dimensional skeletal structure. Therefore, the dispersibility of the fibers in the slurry significantly influences the microstructure and various physical properties of the porous materials. Figure 5 shows the surface and cross-sectional microstructures of SiO_2_ fibrous porous materials with different SHMP contents. As shown in Figure 5a,b,e,f, when the SHMP content was 0.2 wt% and 0.6 wt%, the porous materials contained a large number of fiber bundles, resulting in an uneven distribution of pores in the structure. As shown in Figure 5c,d, when the SHMP content was 0.4 wt%, the porous materials did not contain obvious fiber bundles, and the fibers overlapped each other to form uniformly distributed three-dimensional pores. According to the analysis results in the previous section, when the SHMP content was 0.4 wt%, the quartz fibers exhibited superior dispersibility in the slurry without entanglement and agglomeration. Therefore, the porous materials prepared with this slurry exhibited the best three-dimensional skeleton structure.

**Figure 6:**
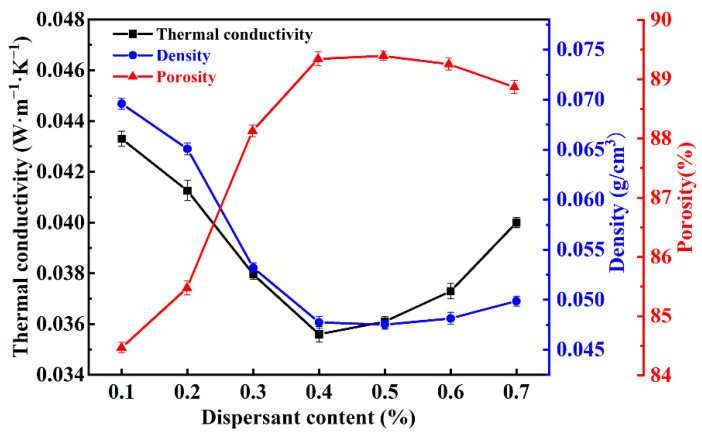
Density, porosity, and thermal conductivity of SiO_2_ fibrous porous materials with different SHMP contents.

The density, porosity, and thermal conductivity of SiO_2_ fibrous porous materials with different SHMP contents are shown in Figure 6. When the SHMP content increased from 0.1 to 0.4 wt%, the porosity increased from 84.46% to 89.39%. However, as the SHMP content further increased to 0.7 wt%, the porosity slightly decreased with an increase in SHMP content. According to the SEM analysis results, the SiO_2_ fibrous porous materials with 0.4 wt% SHMP content had uniformly distributed three-dimensional pores, which endowed the materials with higher porosity. In contrast, the density first decreased from 0.069 g/cm^3^ to 0.048 g/cm^3^ with increasing SHMP content and then slightly increased. The thermal conduction process in fibrous porous materials consists of two components: solid conduction (fiber skeleton) and gas conduction (pores). For a given solid content, gas conduction plays a central role in the total thermal conduction process of porous materials. When fibers disperse uniformly in the slurry, porous materials with ideal three-dimensional skeletal structures have higher porosity, which endows the materials with lower thermal conductivity. Thus, when the SHMP content increased from 0.1 to 0.4 wt%, the thermal conductivity decreased from 0.0433 W·m^−1^·K^−1^ to 0.0356 W·m^−1^·K^−1^, and as the SHMP content further increased to 0.7 wt%, the thermal conductivity increased slightly. Compared with porous materials prepared using low-aspect fibers [10,31], SiO_2_ fibrous porous materials exhibited a lower density (0.0475 g/cm^3^) and thermal conductivity (0.0356 W·m^−1^·K^−1^), providing a new idea for the development of thermal insulation materials.

**Figure 7:**
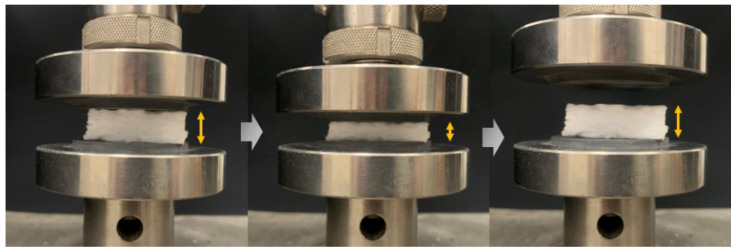
Image of the compression test for the SiO_2_ fibrous porous material under 50% applied strain (resilience ratio > 95%).

**Figure 8:**
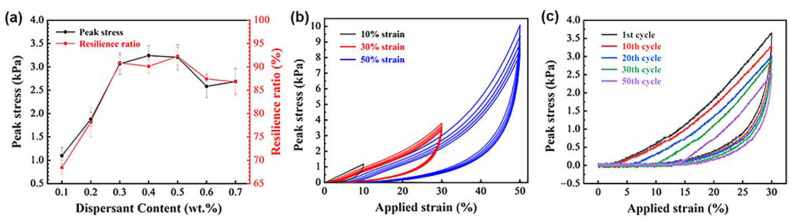
(**a**) Peak stress and resilience ratio of SiO_2_ fibrous porous materials with different SHMP contents at 30% applied strain after 5 compression cycles. (**b**) Compressive rebound curves of SiO_2_ fibrous porous materials under different applied strains for 5 cycles. (**c**) Compressive rebound curves of SiO_2_ fibrous porous materials under 30% applied strain for different cycles.

In addition, the use of high aspect ratio fibers is also conducive to improve the elasticity of porous materials. As shown in Figure 7, the SiO_2_ fibrous porous materials in this experiment can withstand a high applied strain and can recover their original volume after the release of the stress. Figure 8a shows the peak stresses and resilience ratio of SiO_2_ fibrous porous materials with different SHMP contents at 30% applied strain after five compression cycles. The peak stress and resilience ratio increased and then slightly decreased with an increase in SHMP content. When the porous material was compressed, the pores were deformed due to fiber bending, leading to a reduction in the volume of pores, which further resulted in pores that were densely packed and almost parallel to each other. The structural densification led to porous materials with high peak stress. When the pressure was released, the pores returned to their original volume; thus, the porous materials recovered their original shape. However, the resilience ratio may not reach 100% because some pores were destroyed and lost deformability. As the SHMP content increased from 0 to 0.4 wt%, the porosity of the three-dimensional skeleton structure increased, and the distribution of fiber lap joints became more uniform. The uniformly distributed lap joints shared the applied pressure, preventing the pores from being destroyed, which resulted in an increase in the peak stress and resilience ratio of porous materials. As the SHMP content further increased from 0.4 to 0.7 wt%, the porosity decreased, and the peak stress and resilience ratio of porous materials also decreased. Therefore, the SiO_2_ fibrous porous materials with an SHMP content of 0.4 wt% exhibited the best elasticity with a peak stress of 3.24 kPa and resilience ratio of 91.11%.

**Figure 9:**
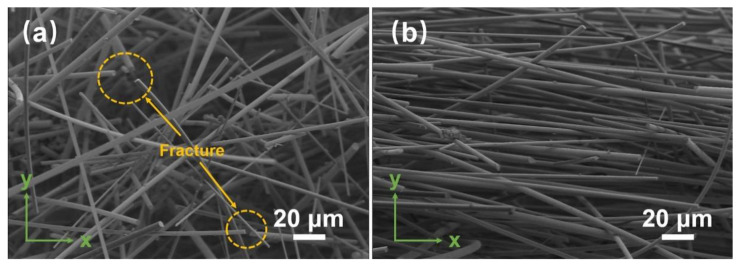
SEM images of SiO_2_ fibrous porous ceramics with 0.4 wt% SHMP content under different applied strains. (**a**) 30% and (**b**) 50%.

The SiO_2_ fibrous porous materials with SHMP content of 0.4 wt% were subjected to compressive rebound tests at different strains. As shown in Figure 8b, when the applied strain was 10%, the peak stress of the porous materials remained at approximately 1.17 kPa for each cycle, and the resilience ratio was 100% after five cycles of compression. When the applied strain increased to 30%, the resilience ratio of the porous material decreased from 100% to 95.45% with an increasing cycle number, exhibiting a slight plastic deformation. The peak stress of the first cycle increased to 3.78 kPa with an increase in applied strain, leading to the fracture of some fibers in the sample (Figure 9a), which affected the elasticity of the porous material. When the applied strain increased to 50%, the pores were irreversibly deformed due to fiber fracture, resulting in the collapse of the three-dimensional skeletal structure (Figure 9b). As the cycle number increased, the peak stress decreased from 10.08 to 8.21 kPa, and the resilience ratio decreased significantly from 90.88% to 68.67%. Thus, the SiO_2_ fibrous porous materials could not maintain elasticity at 50% of the applied strain. The SiO_2_ fibrous porous materials with SHMP content of 0.4 wt% were subjected to compressive rebound tests for 50 cycles at 30% applied strain (Figure 8c). As the cycle number increased, the peak stress and resilience ratio of the porous materials decreased. When the cycle number reached 30, the resilience ratio remained above 80%, and the peak stress was 78.81% of its initial peak stress, indicating that the porous material still exhibited elasticity. However, when the cycle number was further increased to 50, the resilience ratio of the porous material was only 63.52%, and the peak stress was 69.33% of its initial peak stress. Thus, the porous materials no longer exhibited elasticity due to fiber fracture and pore deformation. Dong et al. [32] prepared fibrous porous materials with low densities (0.560–0.595 g/cm^3^) and low thermal conductivities (0.157 W·m^−1^·K^−1^) by the infiltration method. However, the fibrous materials could just withstand 7–8% strain. In comparison, the SiO_2_ fibrous porous materials exhibited lower density (0.04751 g/cm^3^), lower thermal conductivity (0.0356 W·m^−1^·K^−1^) and better excellent elasticity (withstanding 30% strain).

### 3.3. Effect of Heat Treatment

**Figure 10:**
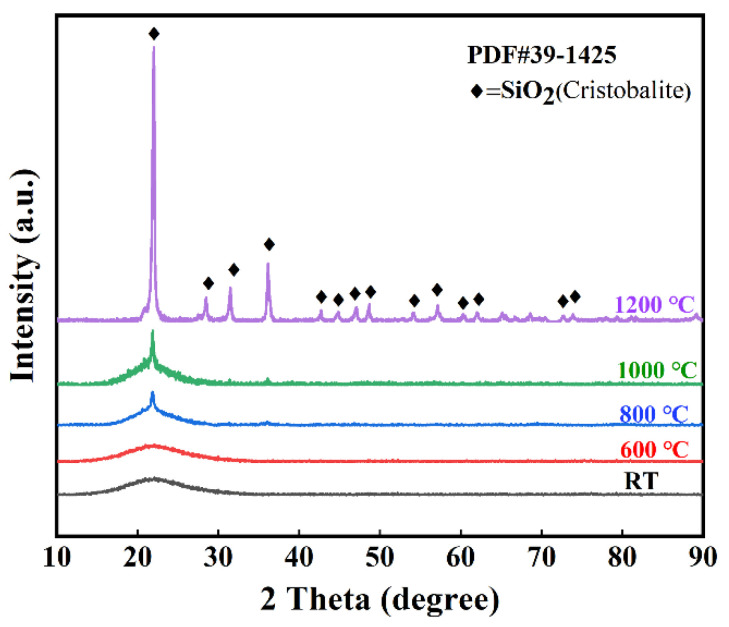
X-ray diffraction patterns of SiO_2_ fibrous porous ceramics before and after heat treatment at different temperatures.

**Figure 11:**
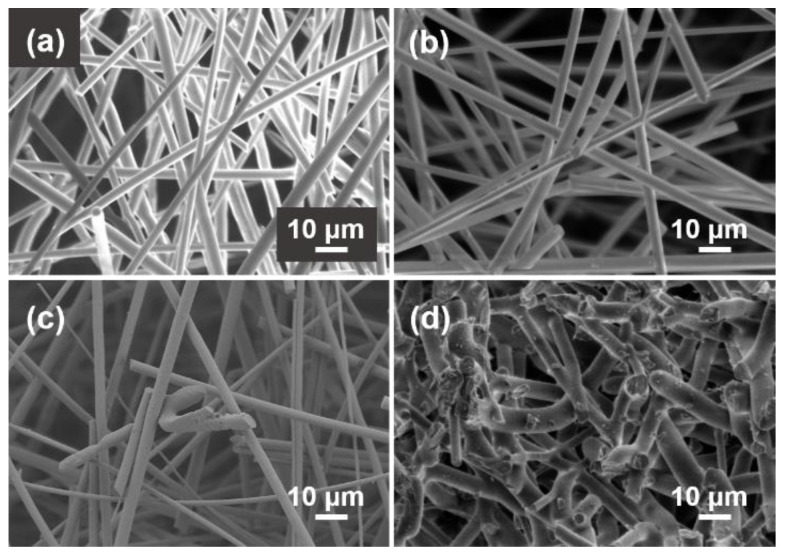
SEM images of SiO_2_ fibrous porous materials after heat treatment at different temperatures. (**a**) 600 °C, (**b**) 800 °C, (**c**) 1000 °C and (**d**) 1200 °C.

Figure 10 shows the X-ray diffraction patterns of SiO_2_ fibrous porous materials after heat treatment at different temperatures. Before and after heat treatment at 600 °C, the wide background at approximately 23° indicated that the main composition of the porous material was amorphous silica. As the heat treatment temperature was increased to 800 °C, the characteristic diffraction peaks of cristobalite (PDF#39-1425) were observed and became increasingly obvious with increasing temperature. When the heat treatment temperature increased to 1200 °C, all the amorphous silica in the porous material transformed into cristobalite. Figure 11 shows the microstructure of SiO_2_ fibrous porous materials after heat treatment at different temperatures. When the heat treatment temperature was 600 °C and 800 °C, the surface of the quartz fibers was smooth, and the porous material still had an ideal three-dimensional skeleton structure (Figure 11a,b). When the heat treatment temperature was 1000 °C, a few quartz fibers were fractured (Figure 11c). According to the XRD analysis results, this was due to the fiber embrittlement caused by the crystallization. When the temperature reached 1200 °C, all the quartz fibers were fractured and deformed due to crystallization and hot softening, leading to the increase in density and decrease in porosity, which resulted in a destruction of the three-dimensional skeletal structure of the porous materials (Figure 11d).

**Figure 12:**
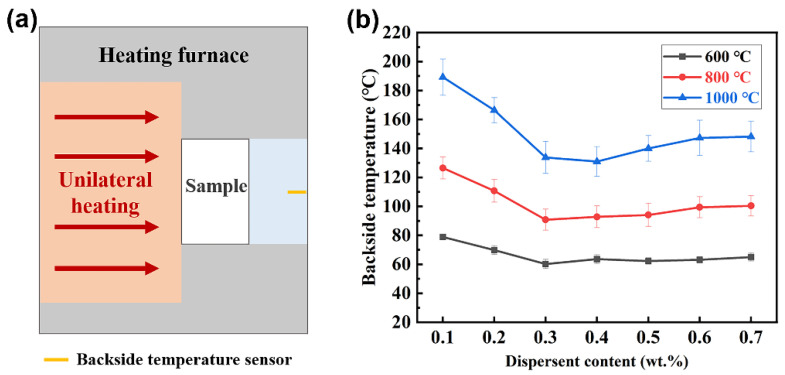
(**a**) Testing principle of the single-surface heating furnace. (**b**) Backside temperatures of SiO_2_ fibrous porous materials with different SHMP contents.

In this experiment, the furnace shown in Figure 12a was used for single-surface heating of porous materials, and the thermal insulation performance of porous materials was evaluated by comparing the backside temperature of the samples at the same heat treatment temperature. Figure 12b shows the backside temperatures of porous materials with different SHMP contents after single-surface heating at different temperatures for 2 h. When the single-surface heating temperature was 600 °C and 800 °C, due to the inherent thermal stability of SiO_2_ fibrous porous materials, the backside of almost all porous materials remained at a relatively low temperature below 100 °C. When the single-surface heating temperature rose to 1000 °C, the backside temperature decreased and then rebounded with an increase in SHMP content, reaching a minimum value of 140 °C when the SHMP content was 0.4 wt%. The change in the backside temperature was opposite to that of porosity with SHMP content and was the same as that of thermal conductivity with SHMP content. These results indicated that SiO_2_ fibrous porous material is an excellent thermal insulation material due to its high porosity and low thermal conductivity of the three-dimensional skeleton structure.

**Figure 13:**
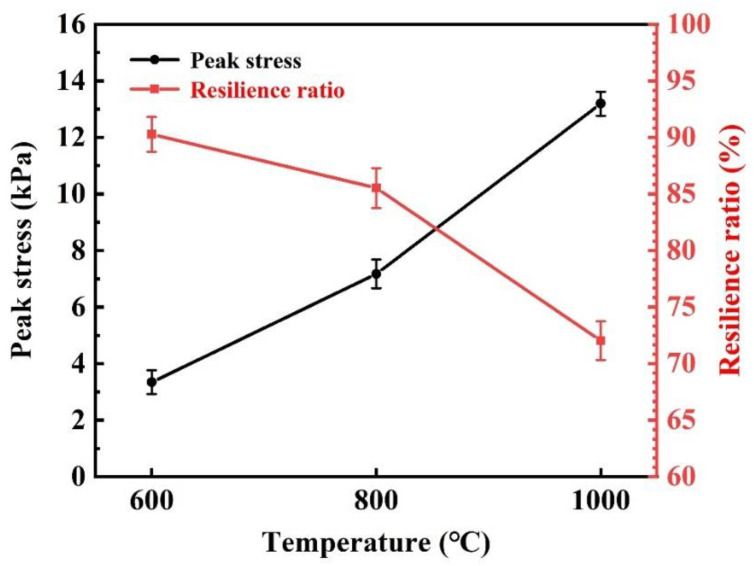
Peak stress and resilience ratio of SiO_2_ fibrous porous materials after compression for 5 cycles under 30% stress at different heat treatment temperatures.

Figure 13 shows the peak stress and resilience ratio of SiO_2_ fibrous porous materials with an SHMP content of 0.4 wt% after heat treatment at different temperatures. It can be observed that the properties (3.35 kPa peak stress and 90.27% resilience ratio) of porous materials after heat treatment at 600 °C are close to those of porous materials at room temperature, indicating that SiO_2_ fibrous porous materials still exhibited excellent elasticity after heat treatment at 600 °C. When the heat treatment temperature was 800 °C, the resilience ratio of porous materials remained above 80%. When the heat treatment temperature rose to 1000 °C, the peak stress of porous materials increased to 13.19 kPa. This is due to the transformation in the fibers from amorphous quartz to cristobalite, which decreased the flexibility and increased the strength of the single fiber. As a result, the load-bearing capacity of the three-dimensional skeleton structure increased with heat treatment temperature, and the peak stress increased. Conversely, the resilience ratio of porous materials decreased to 72.05% at 1000 °C. With an increase in the heat treatment temperature, more fibers fractured, which eventually led to the destruction of the three-dimensional skeletal structure. Therefore, the elasticity of SiO_2_ fibrous porous materials decreased significantly after heat treatment at 1000 °C.

## 4. Conclusions

In order to improve the elasticity of fibrous porous materials, we innovatively reported that a unique three-dimensional skeleton structure was constructed by adjusting the dispersion of fibers in the slurry. The lightweight, thermal insulating and elastic SiO_2_ fibrous porous material was then prepared by compression molding method. In this work, SHMP was used as a dispersant for quartz fibers, and the effect of SHMP content on the dispersibility of fibers in the slurry was investigated. The results show that the Stern layer of quartz fibers adsorbed more negative charges when the anionic dispersant SHMP was added to the slurry, leading to the expansion of the electrical double layer between adjacent fibers, which resulted in an increase in the absolute value of the zeta potential. However, after SHMP reached a critical content of 0.4 wt%, the increase in the number of counter ions led to a compression of the electrical double layer, which resulted in a decrease in the absolute value of the zeta potential. Thus, the slurry with 0.4 wt% SHMP content exhibited a superior dispersibility of fibers.

Moreover, the SHMP content affected the dispersibility of fibers and further affected the microstructure of the three-dimensional skeletal structure formed by the overlapped fibers. Porous materials with 0.4 wt% SHMP content exhibited a higher porosity (89.39%), but a lower density (0.04751 g/cm^3^) and thermal conductivity (0.0356 W·m^−1^·K^−1^). The flexibility of the quartz fibers and the three-dimensional skeleton structure formed by the overlap of fibers with a high aspect ratio endowed porous materials with excellent elasticity. SiO_2_ fibrous porous materials could undergo large strains of 30% and achieved a resilience ratio of 81.69% under the 30th compression cycle. Moreover, SiO_2_ fibrous porous materials exhibited excellent properties after high temperature heat treatment. The materials maintained good elasticity with a resilience ratio of 90.27% after a heat treatment at 600 °C, and the resilience ratio was more than 80% after a heat treatment at 800 °C. However, after a heat treatment at 1000 °C, the elasticity of the material decreased significantly due to fiber crystallization. Because of outstanding thermal insulation and elasticity, SiO_2_ fibrous porous materials could be widely used as thermal insulation materials in the thermal protection systems of spacecraft, petrochemical equipment and various industrial furnaces.

## Data Availability

Not applicable.
